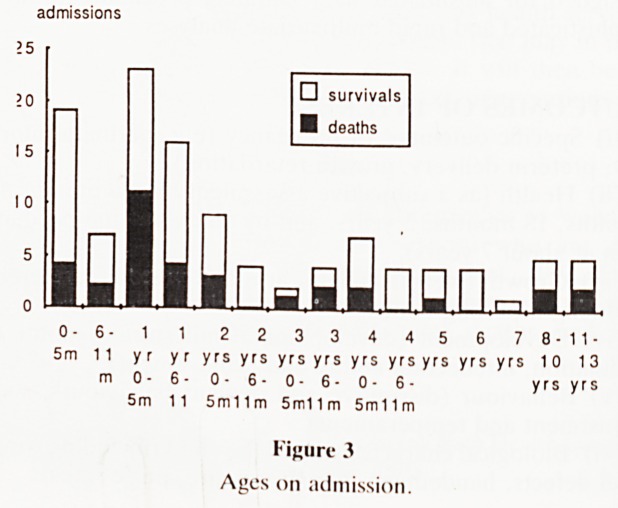# Children of the Nineties

**Published:** 1990-09

**Authors:** Jean Golding

**Affiliations:** Reader in Child Health, University of Bristol

## Abstract

A survey to assess factors, especially those in the environment, which influence child health and development is shortly to be started within the South West region. This study will start during pregnancy and involve both the mother and her partner as well as the child. Data will be collected on environmental exposures, and psychosocial aspects of the family home. Biological samples will also be kept and stored as a means of identifying pollutants inter alia, and to assess their influence on the fetus/infant. The children will be followed up to the age of 7 when they will receive a full educational, psychological and medical examination. Follow up of the outcome of pregnancy and the development of the child will help identify ways in which the environment influences child health and development.


### Children of the Nineties

### A Longitudinal Study of Pregnancy and Childhood Based on the Population of Avon (ALSPAC)

**Jean Golding**
Reader in Child Health, University of Bristol

### SUMMARY
A survey to assess factors, especially those in the environment, which influence child health and development is shortly to be started within the South West region. This study will start during pregnancy and involve both the mother and her partner as well as the child. Data will be collected on environmental exposures, and psychosocial aspects of the family home. Biological samples will also be kept and stored as a means of identifying pollutants inter alia, and to assess their influence on the fetus/infant. The children will be followed up to the age of 7 when they will receive a full educational, psychological and medical examination. Follow up of the outcome of pregnancy and the development of the child will help identify ways in which the environment influences child health and development.

### INTRODUCTION
Health for all children is an admirable aim. In the developing world the way forward is obvious given that the effective introduction of safe water, improved sanitation, nutrition and vaccination are likely to have substantial beneficial effects on the survival, growth and development of children. In the UK the strategies for health improvement are clearly less tangible; as stated in the 1988 volume of 'On the State of Public Health', "activity of health services alone can be expected to have only a limited impact on much of the sickness and premature mortality which occurs in the population . . . many illnesses have their origins in social behaviour, psychological problems and environmental factors" (1).

In the 1989 volume, Sir Donald Acheson states: "Greater understanding of child health and development, and the events of prenatal life will much depend on the study of genetic influences and their interaction with environmental ones – physical, chemical and infective agents, including those of the intrauterine environment, and psychosocial factors. There will be a need for carefully conducted population studies of pregnancy, birth and childhood to attempt to unravel these and interactions. Without such knowledge, the most effective ways of intervening to prevent and treat illness in childhood will remain unclear. Given the tools of multivariate analysis and the rapidly growing availability of specific genetic markers, a new approach lies within our grasp" (2).

A broad, multi-disciplinary research effort is needed to make such measurements and to assess their long-term effects on health. Much has been done in the past looking at individual features predictive of childhood mortality and morbidity such as parental age, social status, unemployment and household size. There has been little attempt to further refine these associations or study the way in which different features of the environment and genetic composition of the child interact to affect his or her health and development.

Retrospective ascertainment of many features that may be aetiologically related to health and development in childhood are prone to problems of biased recall or recording. This, in particular, is true of features of the parental background such as personality and behaviour, attitudes and social support; however it also applies to more objective measures such as health behaviour, infections and drug ingestion. If progress is to be made into a better understanding and promotion of children's health in this country it is essential that a *prospective* study be mounted, with key data collected from early pregnancy onwards.

### WHAT IS ALSPAC?
ALSPAC is an in-depth prospective and longitudinal study of children and parents starting in pregnancy and monitoring health and development from fetal life, through infancy into childhood and the early school years. The cohort comprises all babies born over a 12 month period in the three health districts* of Avon. From the results of the pilot studies it is expected that 95% of the child-bearing women and their families will agree to take part, giving a total of approximately 11,000.

As women and their partners are recruited to the study soon after the confirmation of pregnancy, details of their social background, attitudes towards health care, and psychological well-being will be obtained by self-completion questionnaires. The clinical course of pregnancy and childbirth will be recorded from doctors' case notes. The mothers and fathers will be followed up at 4–6 weeks, 6 months, 18 months, 3 years and 7 years after delivery. Independently assessed details of childhood development, illness, accidents and treatment will be obtained from the case notes of the relevant health workers. When the children are 7 they will undergo a comprehensive medical, intellectual, educational and psychological assessment.

This study will provide the core material for a number of additional cross-sectional and longitudinal research projects designed to be integrated with the main study. Maternal blood and urine will be collected during pregnancy, cord blood and placentae at delivery, maternal blood and, where appropriate, a sample of breast milk postpartum. We are planning to carry out a series of genetic studies to ascertain the way in which the genes of the mother and her child interact with environmental factors to produce disordered health in some children but not in others. Other aspects of the study will include an assessment of exposure to ionising radiation in the home and the relationship between immunology, atopy and environmental exposure to allergens.

### AIMS OF THE STUDY
To determine which environmental, social, psychological, biological and genetic factors are associated with the survival and health of the fetus, infant and child.

Although the study will not itself make any health service provision, it is designed to identify strategies that may in the future serve to improve children's health. It will then be a separate task to put such strategies or health interventions to the test.

### STUDY DESIGN
The cohort sample will comprise all pregnancies to women resident within a geographical area (Avon) who are delivered between April 1st, 1991 and March 31st, 1992. The mothers will be contacted as early in pregnancy as possible. Both livebirths and early and late fetal deaths will be included.

* This comprises the County of Avon, excluding Bath Health District which is in Wessex Regional Health Authority.

Avon includes both urban and rural areas, inner city deprivation, high rise council estates and leafy suburbs. It has a stable population, with little migration out of the area (<2% p.a.), and is demographically representative of Britain as a whole. In order to reduce bias, as well as cost, it will be the parents themselves who will provide most of the information by means of self-completion questionnaires. Validation exercises have been used to select optimal questionnaire design and to check the reliability of the data. In cases of non-response, interviewers will help the mother to complete the questionnaires. Objective measures of child health (clinical records) and development (e.g. health visitor screening) will be used wherever possible or appropriate. Data on specific medical problems will be ascertained from medical records.

The data will be collected as cross-sectional sweeps which may be analysed as cross-sectional data sets and be of value in their own right. The main strength of the study, however, lies in the longitudinal nature of the data that will be obtained.

The progress of the study is fed back to the participants. There will be a newsletter to the parents and birthday cards for the children. Other newsletters will be sent to local health professionals and our collaborators.

### SUB-SAMPLE STUDY

A random 10% of the population will be chosen for closer study. Psychologists will carry out in depth interviews, parenting behaviour will be observed, children will be examined and features of the household environment assessed. This will provide validation for some of the self-reported items and add important detail to others.

### UNIQUE FEATURES

This study will be unique in a number of ways.

(a) It will be the first large longitudinal British study to be designed as such (the national surveys started as birth surveys and only later became longitudinal).

(b) It will be the first geographically based population study commencing in pregnancy to take place in Britain.

(c) It will be the first geographically-based population study including information on the personalities, behaviour and attitudes of both mother and father.

(d) Maternal antenatal and infant blood samples will make it possible for the first time to identify biochemical features of the fetal environment, evidence of microbial or allergen exposure.

(e) Prospectively identified measures of the home environment will include the presence of damp and mould and natural radiation levels.

(f) It will offer the potential for identifying the relationships between the child's genetic endowment and the environment in causing or protecting against disease.

(g) Unlike the British national studies, ALSPAC is designed for automated data handling procedures and for sophisticated and rapid multivariate analyses.

### OUTCOMES OF INTEREST

(i) Specific outcomes of pregnancy (e.g. perinatal mortality, preterm delivery, growth retardation).

(ii) Health (as a subjective assessment by the mother at 6 months, 18 months, 3 years, and by an examining paediatrician at about 7 years).

(iii) Growth (weight, height, arm and head circumference and length of long bones).

(iv) Development (developmental milestones, motor co-ordination, intellectual ability and achievement).

(v) Behaviour (disruptive and deviant behaviours, social adjustment and temperament).

(vi) Biological characteristics of the child (including congenital defects, handedness, blood pressure, pulse rates).

(vii) Morbidity (specific disorders defined as often as possible by signs and symptoms but also including the results of diagnostic tests; such disorders to include eczema, glue ear, specific childhood infections, vaccine reactions, convulsive disorders, chronic bronchitis and asthma, as well as problems resulting in hospital admission and referral).

(viii) Defects of vision, hearing and speech.

(ix) Accidents (with history of what happened, where, resulting injuries and referral to health services).

### FACTORS TO BE ASSESSED

The outcomes listed above will be analysed in relation to a number of factors assessed at various points in time. These will include:

(a) The fetal environment: drugs taken by mother during pregnancy, maternal occupational exposure, maternal infections; development of various antenatal complications such as hypotension, hypertension, vomiting and bleeding; maternal activity during pregnancy, weight gain, and exposure to social drugs (cigarettes, alcohol, caffeine, cannabis).

(b) Changes in the child's environment during the first months of life: onset of maternal depression, household moves, separation from the mother, separation from the father, acute or chronic illness in the household, use of creche, nanny or other child minder.

(c) The physical environment: housing, type of neighbourhood, equipment in the home, presence of damp, type of heating, noise, air pollution levels in the neighbourhood, radon levels.

(d) Parental characteristics: parental ages, family size, past obstetric history, inter-birth intervals, parental heights and weights, medical history of family members.

(e) Social factors: social class, parental education, single parent status, changes in partner.

(f) Psychological factors: personality measures, depression and anxiety.

(g) Psychosocial environment: including items such as social support, marital relationship, sibling relationships.

(h) Health behaviour: attendance for antenatal care, attempt at and duration of breast feeding and weaning practice, taking the child for immunisations, and dental check-ups.

(i) Attitudes: The mother's attitude towards her own and her child's health care, the partner's attitude towards the mother and the child.

(j) The genetic composition of mother and child. Cell lines will be established on all members of the cohort so as to provide genetic material in order to test genetic hypotheses relating to specific diseases and variations in growth, development and health.

### CONFIDENTIALITY

Confidentiality has the highest priority in the study. The mothers and fathers are being asked to divulge sensitive information to the research team using self-administered questionnaires. In addition, case notes and laboratory investigation results are being reviewed. All these data are confidential and will not be divulged to any individual, agency or health service.

A Parents' Consultative Body has been established in order to provide advice and information on sensitive matters and to help develop literature for the parents. An Ethics and Law Advisory sub-committee comprising members of the Bristol University departments of Law, Philosophy, Theology, Pharmacology, Obstetrics and Child Health, together with an observer from the Department of Health and an NHS paediatrician is considering various aspects of the study. They have advised the Steering Committee of the need to obtain maternal consent before the biological samples are used for research purposes.

On a few occasions the study will involve the use of an interviewer to obtain information. Knowledge gained by an interviewer will not be used to intervene in any particular family; but in response to severe distress within the family the mother or father may be guided to seek appropriate professional help.

The computer file will not reveal any information directly identifiable with individuals or families. Information gathered during the study will be published in a form in which individuals or families cannot be identified.

### COLLABORATION

The study is designed to identify the different factors influencing child health and development. Collaboration with other research groups or individuals is encouraged, not only within Bristol but nationally and internationally.

Collaborators are invited to devise proposals and write research grant applications for specific projects which interlock within the core framework of the study. The Steering Committee (see below) will assess the appropriateness of applications for collaboration in order to avoid overlap and optimise sample utilisation. Applications for funding are required to include a component towards the cost of maintaining and managing the ALSPAC data files.

### ADMINISTRATION

The ALSPAC study is an initiative of the Division of Epidemiology based in the Institute of Child Health, University of Bristol.

A Steering Committee guides the development of the project and promotes further research plans. A number of scientific and other advisory sub-committees have been set up. The Steering Committee comprises:

Professor J. D. Baum, Institute of Child Health, Bristol.
Dr. Jean Golding (Chair), Institute of Child Health, Bristol.
Professor Catherine Peckham, Institute of Child Health, London.
Professor Marcus Pembrey, Institute of Child Health, London.
Dr. C. Pennock, Depts. Pathology & Child Health, University of Bristol.
Dr. J. I. Pollock (Secretary), Institute of Child Health, Bristol.
Professor Michael Rutter, Institute of Psychiatry, London.
Professor G. M. Stirrat, Dept. Obstetrics & Gynaecology, University of Bristol.

### REFERENCES

1. D.H.S.S. On the State of the Public Health for the Year 1987. H.M.S.O. London 1988, p. 4.
2. D.H.S.S. On the State of the Public Health for the Year 1988. H.M.S.O. London 1989, p. 73.

**Further information** may be obtained from: Dr. J. Golding, Division of Epidemiology, Institute of Child Health, University of Bristol (Tel. (0272) 225967).

*Continued from opposite page.*

**Figure 1**

Admissions for HIV by half-years, July 1986–Nov 1989.

**Figure 2**

Complaints presented on admission.

**Figure 3**

Ages on admission.

## Figures and Tables

**Figure 1 f1:**
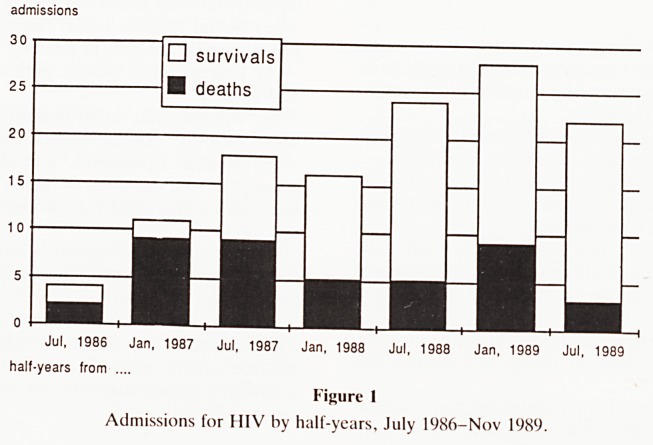


**Figure 2 f2:**
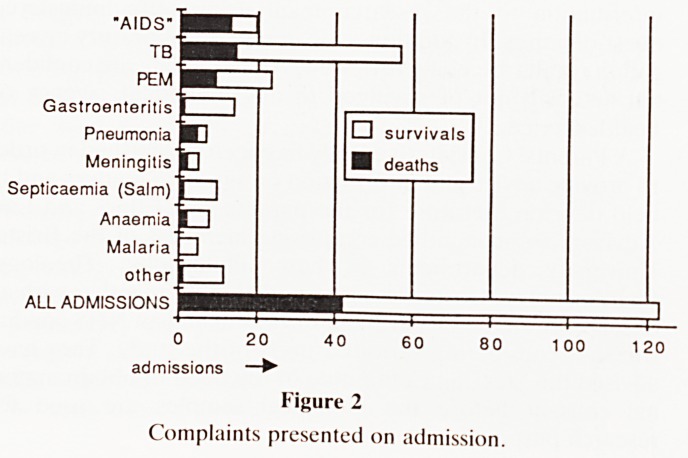


**Figure 3 f3:**